# 1,3-Bis(3-methyl­phen­yl)thio­urea: triclinic polymorph

**DOI:** 10.1107/S1600536809003146

**Published:** 2009-02-06

**Authors:** Durre Shahwar, M. Nawaz Tahir, Muhammad Akmal Khan, Naeem Ahmad, Muhammad Furqan

**Affiliations:** a Department of Chemistry, Government College University, Lahore, Pakistan; b Department of Physics, University of Sargodha, Sargodha, Pakistan

## Abstract

The title compound, C_15_H_16_N_2_S, crystallizes with two molecules in the asymmetric unit. The crystallographic behaviour of the two isomers is different. The mol­ecules are dimerized, forming an *R*_2_^2^(8) ring motif due to inter­molecular N—H⋯S hydrogen bonds. C—H⋯S hydrogen bonds form *R*_2_^2^(12) ring motifs. In one molecule, the dihedral angle between the benzene rings is 62.54 (6)°, whereas in the other it is 79.54 (6)°. The H atoms of one of the methyl groups in each molecule are disordered over two sites, with occupancy ratios of 0.52 (3):0.48 (3) and 0.60 (3):0.40 (3).

## Related literature

For general background, see: Chen *et al.* (2006 ▶). For a report of the title compound in the monoclinic crystal system, see: Soriano-Garcia *et al.* (2003 ▶). For graph-set notation, see: Bernstein *et al.* (1995 ▶).
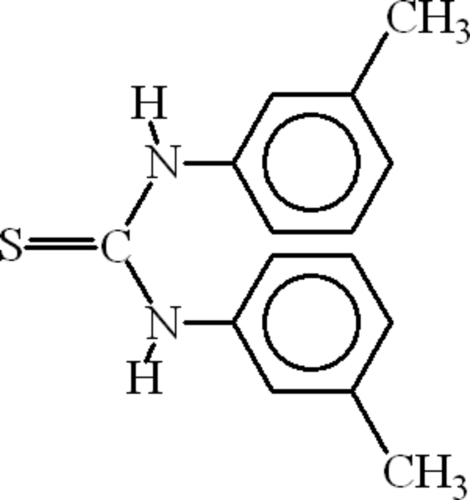


## Experimental

### 

#### Crystal data

C_15_H_16_N_2_S
*M* *_r_* = 256.36
Triclinic, 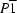

*a* = 10.0483 (5) Å
*b* = 12.0993 (7) Å
*c* = 13.1100 (7) Å
α = 67.633 (2)°
β = 73.496 (1)°
γ = 74.994 (2)°
*V* = 1392.64 (13) Å^3^
*Z* = 4
Mo *K*α radiation radiation
μ = 0.22 mm^−1^
*T* = 296 (2) K
0.25 × 0.14 × 0.10 mm

#### Data collection

Bruker Kappa APEXII CCD diffractometer
Absorption correction: multi-scan (*SADABS*; Bruker, 2005 ▶) *T*_min_ = 0.960, *T*_max_ = 0.982
31605 measured reflections
7452 independent reflections
4540 reflections with *I* > 2σ(*I*)
*R*_int_ = 0.042

#### Refinement

*R*[*F* ^2^ > 2σ(*F* ^2^)] = 0.047
*wR*(*F* ^2^) = 0.116
*S* = 1.00
7452 reflections
332 parameters
H-atom parameters constrained
Δρ_max_ = 0.30 e Å^−3^
Δρ_min_ = −0.26 e Å^−3^

### 

Data collection: *APEX2* (Bruker, 2007 ▶); cell refinement: *SAINT* (Bruker, 2007 ▶); data reduction: *SAINT*; program(s) used to solve structure: *SHELXS97* (Sheldrick, 2008 ▶); program(s) used to refine structure: *SHELXL97* (Sheldrick, 2008 ▶); molecular graphics: *ORTEP-3 for Windows* (Farrugia, 1997 ▶) and *PLATON* (Spek, 2003 ▶); software used to prepare material for publication: *WinGX* (Farrugia, 1999 ▶) and *PLATON*.

## Supplementary Material

Crystal structure: contains datablocks global, I. DOI: 10.1107/S1600536809003146/at2714sup1.cif

Structure factors: contains datablocks I. DOI: 10.1107/S1600536809003146/at2714Isup2.hkl

Additional supplementary materials:  crystallographic information; 3D view; checkCIF report

## Figures and Tables

**Table 1:** Hydrogen-bond geometry (Å, °)

*D*—H⋯*A*	*D*—H	H⋯*A*	*D*⋯*A*	*D*—H⋯*A*
N1—H1⋯S2^i^	0.86	2.46	3.2528 (16)	154
N4—H4*A*⋯S1^i^	0.86	2.63	3.4856 (16)	172
C2—H2⋯S1^ii^	0.93	2.83	3.7492 (19)	168

Symmetry codes: (i) 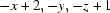; (ii) 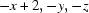.

**Fig. 1.**
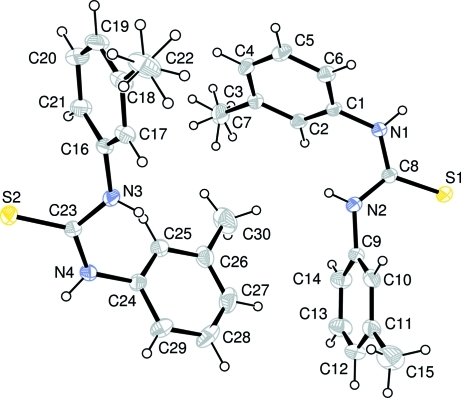
ORTEP drawing of the title compound with the atom numbering scheme. The thermal ellipsoids are drawn at the 30% probability level. H atoms are shown by small circles of arbitrary radii.

**Fig. 2.**
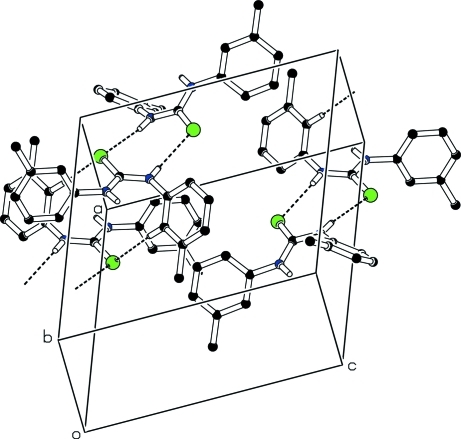
The partial packing figure (PLATON: Spek, 2003) which shows that intermolecular H-bonds form the ring motifs.

## supplementary crystallographic information

### Comment

Bisthiourea analogs have attracted wide attention in recent years on account of their versatile applications ranging; catalysis, biological activity and sophisticate optical technology. Their Palladium complexes have been reported as efficient catalysts for Heck and Suzuki coupling reactions (Chen *et al.*, 2006). The title compound (I), (Fig. 1) is one of the series of bisthioureas synthesized for further complexation and organic derivatization.

Soriano-Garcia *et al.* (2003) has published the crystal structure of the title compound with monoclinic crystal system. In the reported structure the methyl groups are oriented in *cis* form. The title compound (I) crystallizes in triclinic crystal system with two chemically equivalant asymmetric units having minor differences in bond distances and bond angles. In this compound the methyl groups are almost in *trans* forms. The major difference in the two chemical units of (I) is of the dihederal angles between the benzene rings. The ring A (C1–C6) is oriented at an angle of 62.54 (6)° with the ring B (C9–C14) in one molecule, whereas the same between the ring C (C16–C21) and ring D (C24–C29) is 79.54 (6)°. Although the moieties around S═O bonds are similar as far as chemistry is concerned, but they behave differently in forming ring motifs. There exist only intermolecular H-bonds (Table 1). The hydrogen bonds of type N—H···S form *R*_2_^2^(8) (Bernstein *et al.*, 1995), whereas H-bonds of type C—H···S make *R*_2_^2^(12) ring motifs (Fig. 2). The H atoms of one of methyl groups in each chemical units are disordered over two sites with occupancies ratios 0.52 (3):0.48 (3) and 0.60 (3):0.40 (3).

### Experimental

The title compound was prepared by adding CS_2_ dropwise in *m*-toluidine (2 g, 0.0187 mol) dissolved in alkaline (NaOH; 0.75 g, 0.0187 mol) ethanol (95% aq.) while stirring continuously at room temperature for one hour. The precipitated product was filtered and recrystallized from warm methanol.

### Refinement

H atoms were positioned geometrically, with N—H = 0.86 Å, C—H = 0.93 and 0.96 Å for aromatic and methyl H, and constrained to ride on their parent atoms, with U_iso_(H) = xU_eq_(C, N), where x = 1.5 for methyl H, and x = 1.2 for all other H atoms.

### Crystal data

**Table:**

C_15_H_16_N_2_S	*Z* = 4
*M**_r_* = 256.36	*F*(000) = 544
Triclinic, *P*1	*D*_x_ = 1.233 Mg m^−^^3^
Hall symbol: -P 1	Mo *K*α radiation radiation, λ = 0.71073 Å
*a* = 10.0483 (5) Å	Cell parameters from 7452 reflections
*b* = 12.0993 (7) Å	θ = 2.5–29.1°
*c* = 13.1100 (7) Å	µ = 0.22 mm^−^^1^
α = 67.633 (2)°	*T* = 296 K
β = 73.496 (1)°	Needle, colourless
γ = 74.994 (2)°	0.25 × 0.14 × 0.10 mm
*V* = 1392.64 (13) Å^3^	

### Data collection

**Table:**

Bruker Kappa APEXII CCD diffractometer	7452 independent reflections
Radiation source: fine-focus sealed tube	4540 reflections with *I* > 2σ(*I*)
graphite	*R*_int_ = 0.042
Detector resolution: 7.40 pixels mm^-1^	θ_max_ = 29.1°, θ_min_ = 2.5°
ω scans	*h* = −13→12
Absorption correction: multi-scan (*SADABS*; Bruker, 2005)	*k* = −16→16
*T*_min_ = 0.960, *T*_max_ = 0.982	*l* = −17→17
31605 measured reflections	

### Refinement

**Table:**

Refinement on *F*^2^	Secondary atom site location: difference Fourier map
Least-squares matrix: full	Hydrogen site location: inferred from neighbouring sites
*R*[*F*^2^ > 2σ(*F*^2^)] = 0.047	H-atom parameters constrained
*wR*(*F*^2^) = 0.116	*w* = 1/[σ^2^(*F*_o_^2^) + (0.0448*P*)^2^ + 0.2934*P*] where *P* = (*F*_o_^2^ + 2*F*_c_^2^)/3
*S* = 1.00	(Δ/σ)_max_ = 0.001
7452 reflections	Δρ_max_ = 0.30 e Å^−^^3^
332 parameters	Δρ_min_ = −0.26 e Å^−^^3^
0 restraints	Extinction correction: *SHELXL97* (Sheldrick, 2008), Fc^*^=kFc[1+0.001xFc^2^λ^3^/sin(2θ)]^-1/4^
Primary atom site location: structure-invariant direct methods	Extinction coefficient: 0.0096 (12)

### Special details

**Table:**

Geometry. Bond distances, angles *etc*. have been calculated using the rounded fractional coordinates. All su's are estimated from the variances of the (full) variance-covariance matrix. The cell e.s.d.'s are taken into account in the estimation of distances, angles and torsion angles
Refinement. Refinement of *F*^2^ against ALL reflections. The weighted *R*-factor *wR* and goodness of fit *S* are based on *F*^2^, conventional *R*-factors *R* are based on *F*, with *F* set to zero for negative *F*^2^. The threshold expression of *F*^2^ > σ(*F*^2^) is used only for calculating *R*-factors(gt) *etc*. and is not relevant to the choice of reflections for refinement. *R*-factors based on *F*^2^ are statistically about twice as large as those based on *F*, and *R*- factors based on ALL data will be even larger.

### Fractional atomic coordinates and isotropic or equivalent isotropic displacement parameters (Å^2^)

**Table:**

	*x*	*y*	*z*	*U*_iso_*/*U*_eq_	Occ. (<1)
S1	1.22719 (4)	0.05439 (4)	−0.03732 (4)	0.0398 (1)	
N1	1.07002 (14)	0.04399 (14)	0.16375 (11)	0.0427 (5)	
N2	0.99190 (16)	0.20068 (14)	0.01812 (12)	0.0535 (5)	
C1	0.95406 (17)	0.07128 (15)	0.24821 (13)	0.0365 (5)	
C2	0.81844 (17)	0.06802 (16)	0.24622 (14)	0.0396 (6)	
C3	0.70482 (17)	0.09124 (16)	0.32960 (15)	0.0405 (6)	
C4	0.73165 (19)	0.11622 (17)	0.41577 (15)	0.0444 (6)	
C5	0.8671 (2)	0.11785 (18)	0.41887 (15)	0.0474 (6)	
C6	0.97971 (18)	0.09560 (16)	0.33493 (14)	0.0424 (6)	
C7	0.55815 (10)	0.08570 (18)	0.32691 (8)	0.0637 (8)	
C8	1.08837 (9)	0.10228 (10)	0.05234 (9)	0.0352 (5)	
C9	0.98934 (9)	0.27754 (9)	−0.09582 (8)	0.0422 (6)	
C10	1.06849 (9)	0.36925 (9)	−0.14583 (9)	0.0462 (6)	
C11	1.06095 (19)	0.44879 (17)	−0.25359 (17)	0.0512 (7)	
C12	0.9708 (2)	0.4346 (2)	−0.30793 (17)	0.0581 (7)	
C13	0.8915 (2)	0.3441 (2)	−0.25806 (19)	0.0642 (8)	
C14	0.9009 (2)	0.26440 (19)	−0.15136 (18)	0.0565 (7)	
C15	1.1498 (3)	0.5470 (2)	−0.3100 (2)	0.0904 (10)	
S2	0.68029 (5)	0.12464 (5)	0.70426 (4)	0.0523 (2)	
N3	0.42439 (14)	0.25189 (14)	0.74526 (11)	0.0406 (5)	
N4	0.54748 (14)	0.18299 (13)	0.88624 (11)	0.0390 (5)	
C16	0.40038 (17)	0.27933 (15)	0.63565 (14)	0.0361 (5)	
C17	0.28808 (18)	0.24203 (16)	0.62503 (15)	0.0417 (6)	
C18	0.2573 (2)	0.27196 (17)	0.51940 (17)	0.0471 (6)	
C19	0.3421 (2)	0.34043 (19)	0.42651 (17)	0.0551 (7)	
C20	0.4540 (2)	0.3772 (2)	0.43720 (16)	0.0582 (7)	
C21	0.48483 (19)	0.34709 (17)	0.54144 (15)	0.0465 (6)	
C22	0.13661 (15)	0.22770 (16)	0.50862 (8)	0.0752 (10)	
C23	0.54422 (10)	0.19099 (9)	0.78139 (10)	0.0342 (5)	
C24	0.45749 (9)	0.25761 (10)	0.94960 (8)	0.0372 (6)	
C25	0.43907 (9)	0.38272 (10)	0.90138 (10)	0.0453 (6)	
C26	0.3547 (2)	0.4579 (2)	0.96132 (18)	0.0560 (8)	
C27	0.2899 (2)	0.4044 (3)	1.0711 (2)	0.0670 (9)	
C28	0.3101 (2)	0.2805 (3)	1.12038 (18)	0.0673 (9)	
C29	0.3942 (2)	0.2051 (2)	1.06051 (15)	0.0513 (7)	
C30	0.3375 (3)	0.5945 (2)	0.9066 (2)	0.0873 (11)	
H1	1.13465	−0.01597	0.18672	0.0513*	
H2	0.80280	0.04998	0.18812	0.0476*	
H2A	0.92505	0.21939	0.06972	0.0641*	
H4	0.65730	0.13211	0.47236	0.0533*	
H5	0.88315	0.13404	0.47786	0.0569*	
H6	1.07096	0.09705	0.33704	0.0509*	
H7A	0.49223	0.11031	0.38666	0.0956*	0.52 (3)
H7B	0.53615	0.13905	0.25571	0.0956*	0.52 (3)
H7C	0.55241	0.00419	0.33664	0.0956*	0.52 (3)
H10	1.12739	0.37773	−0.10687	0.0554*	
H12	0.96368	0.48768	−0.38009	0.0697*	
H13	0.83119	0.33635	−0.29629	0.0771*	
H14	0.84771	0.20238	−0.11741	0.0677*	
H15A	1.17667	0.56008	−0.38948	0.1357*	
H15B	1.09676	0.62071	−0.29742	0.1357*	
H15C	1.23277	0.52300	−0.27894	0.1357*	
H7D	0.56037	0.06751	0.26121	0.0956*	0.48 (3)
H7E	0.52162	0.02354	0.39333	0.0956*	0.48 (3)
H7F	0.49880	0.16251	0.32447	0.0956*	0.48 (3)
H3	0.35614	0.27644	0.79281	0.0488*	
H4A	0.61004	0.12734	0.91816	0.0468*	
H17	0.23211	0.19637	0.68898	0.0500*	
H19	0.32293	0.36201	0.35539	0.0661*	
H20	0.50979	0.42304	0.37329	0.0698*	
H21	0.56112	0.37193	0.54828	0.0557*	
H22A	0.10577	0.27958	0.44028	0.1128*	0.60 (3)
H22B	0.06017	0.22897	0.57199	0.1128*	0.60 (3)
H22C	0.16696	0.14634	0.50670	0.1128*	0.60 (3)
H25	0.48423	0.41720	0.82716	0.0544*	
H27	0.23161	0.45285	1.11249	0.0804*	
H28	0.26660	0.24647	1.19520	0.0807*	
H29	0.40748	0.12129	1.09441	0.0615*	
H30A	0.42045	0.61563	0.85036	0.1314*	
H30B	0.32383	0.63126	0.96287	0.1314*	
H30C	0.25721	0.62321	0.87185	0.1314*	
H22D	0.11703	0.15635	0.57183	0.1128*	0.40 (3)
H22E	0.16127	0.20871	0.44012	0.1128*	0.40 (3)
H22F	0.05461	0.28983	0.50702	0.1128*	0.40 (3)

### Atomic displacement parameters (Å^2^)

**Table:**

	*U*^11^	*U*^22^	*U*^33^	*U*^12^	*U*^13^	*U*^23^
S1	0.0400 (2)	0.0445 (3)	0.0306 (2)	−0.0015 (2)	−0.0038 (2)	−0.0139 (2)
N1	0.0417 (8)	0.0467 (9)	0.0287 (8)	0.0065 (7)	−0.0063 (6)	−0.0104 (7)
N2	0.0526 (9)	0.0506 (10)	0.0310 (8)	0.0121 (7)	0.0005 (7)	−0.0054 (7)
C1	0.0423 (9)	0.0339 (10)	0.0263 (9)	−0.0025 (7)	−0.0044 (7)	−0.0071 (7)
C2	0.0480 (9)	0.0417 (11)	0.0308 (9)	−0.0035 (8)	−0.0109 (7)	−0.0145 (8)
C3	0.0413 (9)	0.0392 (10)	0.0355 (10)	−0.0033 (7)	−0.0061 (7)	−0.0100 (8)
C4	0.0497 (10)	0.0446 (11)	0.0338 (10)	−0.0032 (8)	−0.0004 (8)	−0.0166 (9)
C5	0.0614 (11)	0.0514 (12)	0.0337 (10)	−0.0116 (9)	−0.0078 (8)	−0.0190 (9)
C6	0.0455 (9)	0.0466 (11)	0.0365 (10)	−0.0093 (8)	−0.0094 (8)	−0.0136 (9)
C7	0.0452 (11)	0.0781 (16)	0.0662 (15)	−0.0067 (10)	−0.0091 (10)	−0.0263 (13)
C8	0.0380 (8)	0.0363 (10)	0.0299 (9)	−0.0057 (7)	−0.0061 (7)	−0.0104 (8)
C9	0.0407 (9)	0.0372 (10)	0.0350 (10)	0.0023 (8)	−0.0045 (7)	−0.0053 (8)
C10	0.0377 (9)	0.0457 (12)	0.0511 (12)	−0.0015 (8)	−0.0130 (8)	−0.0127 (10)
C11	0.0444 (10)	0.0380 (11)	0.0558 (13)	−0.0039 (8)	−0.0068 (9)	−0.0034 (10)
C12	0.0638 (12)	0.0516 (13)	0.0429 (12)	0.0008 (10)	−0.0172 (10)	−0.0009 (10)
C13	0.0703 (14)	0.0669 (16)	0.0596 (14)	−0.0166 (12)	−0.0275 (11)	−0.0121 (12)
C14	0.0627 (12)	0.0491 (13)	0.0544 (13)	−0.0198 (10)	−0.0118 (10)	−0.0072 (11)
C15	0.0785 (17)	0.0647 (17)	0.102 (2)	−0.0291 (14)	−0.0176 (15)	0.0108 (15)
S2	0.0482 (3)	0.0674 (4)	0.0376 (3)	0.0154 (2)	−0.0142 (2)	−0.0259 (3)
N3	0.0342 (7)	0.0573 (10)	0.0293 (8)	0.0024 (6)	−0.0083 (6)	−0.0186 (7)
N4	0.0404 (7)	0.0451 (9)	0.0316 (8)	0.0042 (6)	−0.0145 (6)	−0.0151 (7)
C16	0.0399 (8)	0.0354 (10)	0.0317 (9)	0.0032 (7)	−0.0125 (7)	−0.0125 (8)
C17	0.0466 (9)	0.0367 (10)	0.0408 (10)	−0.0026 (8)	−0.0165 (8)	−0.0093 (8)
C18	0.0605 (11)	0.0353 (10)	0.0516 (12)	0.0036 (9)	−0.0310 (10)	−0.0149 (9)
C19	0.0727 (13)	0.0548 (13)	0.0370 (11)	0.0057 (10)	−0.0279 (10)	−0.0132 (10)
C20	0.0630 (12)	0.0622 (14)	0.0352 (11)	−0.0087 (10)	−0.0095 (9)	−0.0026 (10)
C21	0.0480 (10)	0.0483 (12)	0.0386 (11)	−0.0088 (9)	−0.0125 (8)	−0.0066 (9)
C22	0.0942 (17)	0.0619 (15)	0.0879 (18)	−0.0124 (13)	−0.0588 (15)	−0.0166 (14)
C23	0.0365 (8)	0.0362 (10)	0.0298 (9)	−0.0048 (7)	−0.0083 (7)	−0.0108 (8)
C24	0.0361 (8)	0.0495 (12)	0.0311 (9)	−0.0035 (7)	−0.0109 (7)	−0.0185 (8)
C25	0.0469 (10)	0.0516 (12)	0.0381 (10)	−0.0008 (8)	−0.0124 (8)	−0.0179 (9)
C26	0.0525 (11)	0.0621 (14)	0.0619 (14)	0.0079 (10)	−0.0208 (10)	−0.0349 (12)
C27	0.0563 (12)	0.0884 (19)	0.0671 (16)	0.0039 (12)	−0.0065 (11)	−0.0526 (15)
C28	0.0688 (14)	0.100 (2)	0.0393 (12)	−0.0276 (14)	0.0088 (10)	−0.0356 (13)
C29	0.0627 (12)	0.0615 (14)	0.0347 (11)	−0.0198 (10)	−0.0067 (9)	−0.0178 (10)
C30	0.105 (2)	0.0608 (16)	0.101 (2)	0.0215 (14)	−0.0380 (16)	−0.0435 (15)

### Geometric parameters (Å, °)

**Table:**

S1—C8	1.6827 (12)	C10—H10	0.9300
S2—C23	1.6734 (13)	C12—H12	0.9300
N1—C8	1.3398 (17)	C13—H13	0.9300
N1—C1	1.428 (2)	C14—H14	0.9300
N2—C8	1.342 (2)	C15—H15A	0.9600
N2—C9	1.4314 (17)	C15—H15C	0.9600
N1—H1	0.8600	C15—H15B	0.9600
N2—H2A	0.8600	C16—C17	1.377 (3)
N3—C16	1.423 (2)	C16—C21	1.383 (3)
N3—C23	1.3476 (19)	C17—C18	1.396 (3)
N4—C23	1.3500 (18)	C18—C19	1.380 (3)
N4—C24	1.4297 (19)	C18—C22	1.506 (3)
N3—H3	0.8600	C19—C20	1.370 (3)
N4—H4A	0.8600	C20—C21	1.378 (3)
C1—C6	1.381 (2)	C24—C29	1.380 (2)
C1—C2	1.381 (3)	C24—C25	1.3851 (17)
C2—C3	1.391 (3)	C25—C26	1.389 (3)
C3—C7	1.503 (2)	C26—C27	1.375 (3)
C3—C4	1.384 (3)	C26—C30	1.515 (3)
C4—C5	1.379 (3)	C27—C28	1.373 (5)
C5—C6	1.387 (3)	C28—C29	1.390 (4)
C9—C14	1.369 (2)	C17—H17	0.9300
C9—C10	1.3787 (15)	C19—H19	0.9300
C10—C11	1.384 (2)	C20—H20	0.9300
C11—C12	1.378 (3)	C21—H21	0.9300
C11—C15	1.504 (3)	C22—H22A	0.9600
C12—C13	1.368 (3)	C22—H22B	0.9600
C13—C14	1.377 (3)	C22—H22C	0.9600
C2—H2	0.9300	C22—H22D	0.9600
C4—H4	0.9300	C22—H22E	0.9600
C5—H5	0.9300	C22—H22F	0.9600
C6—H6	0.9300	C25—H25	0.9300
C7—H7F	0.9600	C27—H27	0.9300
C7—H7E	0.9600	C28—H28	0.9300
C7—H7A	0.9600	C29—H29	0.9300
C7—H7D	0.9600	C30—H30A	0.9600
C7—H7C	0.9600	C30—H30B	0.9600
C7—H7B	0.9600	C30—H30C	0.9600
			
S1···N3^i^	3.4381 (15)	H2A···H2	2.4300
S1···C10	3.6191 (12)	H2A···C1	2.3900
S1···C29^i^	3.518 (2)	H2A···C2	2.5000
S1···N4^ii^	3.4856 (16)	H2A···C30^v^	3.0200
S2···N1^ii^	3.2528 (16)	H2A···H30C^v^	2.4600
S2···C21	3.306 (2)	H3···S1^iv^	3.0800
S1···H2^iii^	2.8300	H3···C10^iv^	2.8200
S1···H4A^ii^	2.6300	H3···C24	2.4500
S1···H3^i^	3.0800	H3···C25	2.6400
S1···H14^iii^	3.1400	H3···H10^iv^	2.5600
S2···H5	3.0700	H4···S2	3.0800
S2···H21	3.0700	H4···C20	3.0900
S2···H1^ii^	2.4600	H4···C21	3.0300
S2···H4	3.0800	H4···H7A	2.3700
N1···S2^ii^	3.2528 (16)	H4A···S1^ii^	2.6300
N2···C2	3.065 (2)	H5···S2	3.0700
N3···S1^iv^	3.4381 (15)	H7A···H4	2.3700
N3···C25	3.078 (2)	H7A···C19	2.9600
N4···S1^ii^	3.4856 (16)	H7B···C28^ix^	3.0300
N2···H2	2.8600	H7B···C29^ix^	3.0200
N3···H25	2.8500	H7C···C17^vi^	2.9200
C2···N2	3.065 (2)	H7D···H2	2.3400
C10···S1	3.6191 (12)	H7E···H7E^vi^	2.5600
C11···C17^i^	3.465 (3)	H7F···C19	2.8500
C12···C17^i^	3.533 (3)	H10···H3^i^	2.5600
C17···C12^iv^	3.533 (3)	H10···H15C	2.4300
C17···C11^iv^	3.465 (3)	H12···H22A^x^	2.5800
C21···S2	3.306 (2)	H12···H22F^x^	2.5600
C21···C21^v^	3.507 (3)	H12···H15A	2.4700
C25···N3	3.078 (2)	H14···S1^iii^	3.1400
C29···S1^iv^	3.518 (2)	H15A···H12	2.4700
C1···H22D^vi^	2.9600	H15B···C5^vii^	3.0200
C1···H2A	2.3900	H15C···H10	2.4300
C2···H22D^vi^	2.9200	H17···H22B	2.4900
C2···H2A	2.5000	H17···H22D	2.4100
C3···H22C^vi^	3.0800	H19···H22A	2.4300
C3···H22D^vi^	3.0700	H19···H22E	2.5100
C4···H22C^vi^	2.9400	H20···H25^v^	2.6000
C5···H22C^vi^	3.0500	H21···S2	3.0700
C5···H15B^vii^	3.0200	H21···C23	2.9900
C8···H2	3.0000	H21···C20^v^	3.0800
C10···H3^i^	2.8200	H21···C21^v^	3.1000
C13···H22B^i^	2.9600	H22A···H19	2.4300
C17···H7C^vi^	2.9200	H22A···H12^x^	2.5800
C19···H7F	2.8500	H22B···C13^iv^	2.9600
C19···H7A	2.9600	H22B···H17	2.4900
C20···H4	3.0900	H22C···C3^vi^	3.0800
C20···H21^v^	3.0800	H22C···C4^vi^	2.9400
C21···H21^v^	3.1000	H22C···C5^vi^	3.0500
C21···H4	3.0300	H22D···C2^vi^	2.9200
C23···H21	2.9900	H22D···H17	2.4100
C23···H25	2.9000	H22D···C1^vi^	2.9600
C24···H3	2.4500	H22D···C3^vi^	3.0700
C25···H3	2.6400	H22E···H19	2.5100
C28···H7B^viii^	3.0300	H22F···H12^x^	2.5600
C29···H7B^viii^	3.0200	H25···N3	2.8500
C30···H2A^v^	3.0200	H25···H20^v^	2.6000
H1···S2^ii^	2.4600	H25···C23	2.9000
H2···N2	2.8600	H25···H30A	2.4300
H2···H7D	2.3400	H27···H30B	2.4800
H2···C8	3.0000	H30A···H25	2.4300
H2···H2A	2.4300	H30B···H27	2.4800
H2···S1^iii^	2.8300	H30C···H2A^v^	2.4600
			
C1—N1—C8	126.62 (14)	H15B—C15—H15C	109.00
C8—N2—C9	126.40 (13)	H15A—C15—H15C	109.00
C1—N1—H1	117.00	C11—C15—H15C	109.00
C8—N1—H1	117.00	C11—C15—H15A	110.00
C8—N2—H2A	117.00	C11—C15—H15B	109.00
C9—N2—H2A	117.00	H15A—C15—H15B	109.00
C16—N3—C23	126.00 (14)	N3—C16—C17	119.09 (16)
C23—N4—C24	126.62 (13)	N3—C16—C21	120.47 (17)
C23—N3—H3	117.00	C17—C16—C21	120.38 (16)
C16—N3—H3	117.00	C16—C17—C18	120.77 (17)
C23—N4—H4A	117.00	C17—C18—C22	120.22 (17)
C24—N4—H4A	117.00	C19—C18—C22	121.77 (17)
N1—C1—C2	120.51 (15)	C17—C18—C19	117.99 (19)
C2—C1—C6	120.26 (16)	C18—C19—C20	121.16 (19)
N1—C1—C6	119.16 (16)	C19—C20—C21	120.84 (19)
C1—C2—C3	121.17 (16)	C16—C21—C20	118.85 (19)
C4—C3—C7	121.46 (16)	N3—C23—N4	115.99 (12)
C2—C3—C7	120.45 (16)	S2—C23—N3	122.89 (10)
C2—C3—C4	118.07 (17)	S2—C23—N4	121.09 (10)
C3—C4—C5	120.90 (18)	N4—C24—C29	119.94 (14)
C4—C5—C6	120.69 (18)	C25—C24—C29	119.95 (14)
C1—C6—C5	118.89 (18)	N4—C24—C25	120.04 (10)
N1—C8—N2	116.15 (12)	C24—C25—C26	121.52 (13)
S1—C8—N2	122.91 (10)	C25—C26—C27	118.0 (2)
S1—C8—N1	120.93 (10)	C25—C26—C30	120.32 (18)
N2—C9—C14	119.50 (14)	C27—C26—C30	121.7 (2)
N2—C9—C10	119.76 (11)	C26—C27—C28	120.9 (3)
C10—C9—C14	120.60 (12)	C27—C28—C29	121.2 (2)
C9—C10—C11	120.50 (12)	C24—C29—C28	118.4 (2)
C10—C11—C15	120.86 (18)	C16—C17—H17	120.00
C10—C11—C12	118.09 (18)	C18—C17—H17	120.00
C12—C11—C15	121.1 (2)	C18—C19—H19	119.00
C11—C12—C13	121.5 (2)	C20—C19—H19	119.00
C12—C13—C14	120.1 (2)	C19—C20—H20	120.00
C9—C14—C13	119.25 (19)	C21—C20—H20	120.00
C1—C2—H2	119.00	C16—C21—H21	121.00
C3—C2—H2	119.00	C20—C21—H21	121.00
C5—C4—H4	120.00	C18—C22—H22A	109.00
C3—C4—H4	120.00	C18—C22—H22B	109.00
C4—C5—H5	120.00	C18—C22—H22C	109.00
C6—C5—H5	120.00	C18—C22—H22D	109.00
C5—C6—H6	121.00	C18—C22—H22E	109.00
C1—C6—H6	121.00	C18—C22—H22F	109.00
C3—C7—H7C	109.00	H22A—C22—H22B	109.00
C3—C7—H7A	109.00	H22A—C22—H22C	109.00
C3—C7—H7B	109.00	H22B—C22—H22C	109.00
C3—C7—H7D	109.00	H22D—C22—H22E	109.00
H7D—C7—H7F	109.00	H22D—C22—H22F	109.00
C3—C7—H7E	109.00	H22E—C22—H22F	109.00
H7D—C7—H7E	109.00	C24—C25—H25	119.00
C3—C7—H7F	109.00	C26—C25—H25	119.00
H7A—C7—H7B	109.00	C26—C27—H27	119.00
H7A—C7—H7C	109.00	C28—C27—H27	120.00
H7B—C7—H7C	109.00	C27—C28—H28	119.00
H7E—C7—H7F	109.00	C29—C28—H28	119.00
C11—C10—H10	120.00	C24—C29—H29	121.00
C9—C10—H10	120.00	C28—C29—H29	121.00
C11—C12—H12	119.00	C26—C30—H30A	109.00
C13—C12—H12	119.00	C26—C30—H30B	109.00
C14—C13—H13	120.00	C26—C30—H30C	109.00
C12—C13—H13	120.00	H30A—C30—H30B	109.00
C9—C14—H14	120.00	H30A—C30—H30C	109.00
C13—C14—H14	120.00	H30B—C30—H30C	109.00
			
C8—N1—C1—C2	59.6 (3)	C10—C9—C14—C13	−0.1 (3)
C8—N1—C1—C6	−123.34 (19)	N2—C9—C14—C13	175.44 (18)
C1—N1—C8—S1	−176.76 (14)	C9—C10—C11—C15	−178.34 (17)
C1—N1—C8—N2	4.4 (2)	C9—C10—C11—C12	1.1 (3)
C9—N2—C8—S1	1.0 (2)	C10—C11—C12—C13	−0.6 (3)
C9—N2—C8—N1	179.79 (14)	C15—C11—C12—C13	178.8 (2)
C8—N2—C9—C10	−83.73 (19)	C11—C12—C13—C14	−0.2 (4)
C8—N2—C9—C14	100.7 (2)	C12—C13—C14—C9	0.5 (3)
C16—N3—C23—S2	−6.7 (2)	N3—C16—C17—C18	177.07 (18)
C16—N3—C23—N4	175.26 (16)	C21—C16—C17—C18	−0.1 (3)
C23—N3—C16—C17	123.74 (19)	N3—C16—C21—C20	−176.69 (19)
C23—N3—C16—C21	−59.1 (3)	C17—C16—C21—C20	0.5 (3)
C23—N4—C24—C25	−50.55 (19)	C16—C17—C18—C19	−0.4 (3)
C24—N4—C23—S2	163.34 (11)	C16—C17—C18—C22	178.32 (18)
C24—N4—C23—N3	−18.5 (2)	C17—C18—C19—C20	0.6 (3)
C23—N4—C24—C29	132.48 (17)	C22—C18—C19—C20	−178.1 (2)
N1—C1—C2—C3	178.28 (17)	C18—C19—C20—C21	−0.2 (4)
C6—C1—C2—C3	1.2 (3)	C19—C20—C21—C16	−0.3 (3)
C2—C1—C6—C5	−0.7 (3)	N4—C24—C25—C26	−178.66 (14)
N1—C1—C6—C5	−177.76 (17)	C29—C24—C25—C26	−1.7 (2)
C1—C2—C3—C4	−0.8 (3)	N4—C24—C29—C28	178.47 (17)
C1—C2—C3—C7	−179.20 (17)	C25—C24—C29—C28	1.5 (3)
C2—C3—C4—C5	−0.1 (3)	C24—C25—C26—C27	0.4 (3)
C7—C3—C4—C5	178.25 (18)	C24—C25—C26—C30	179.50 (18)
C3—C4—C5—C6	0.6 (3)	C25—C26—C27—C28	1.1 (3)
C4—C5—C6—C1	−0.2 (3)	C30—C26—C27—C28	−178.0 (2)
N2—C9—C10—C11	−176.23 (15)	C26—C27—C28—C29	−1.3 (4)
C14—C9—C10—C11	−0.7 (2)	C27—C28—C29—C24	0.0 (3)

Symmetry codes: (i) *x*+1, *y*, *z*−1; (ii) −*x*+2, −*y*, −*z*+1; (iii) −*x*+2, −*y*, −*z*; (iv) *x*−1, *y*, *z*+1; (v) −*x*+1, −*y*+1, −*z*+1; (vi) −*x*+1, −*y*, −*z*+1; (vii) −*x*+2, −*y*+1, −*z*; (viii) *x*, *y*, *z*+1; (ix) *x*, *y*, *z*−1; (x) −*x*+1, −*y*+1, −*z*.

### Hydrogen-bond geometry (Å, °)

**Table:**

*D*—H···*A*	*D*—H	H···*A*	*D*···*A*	*D*—H···*A*
N1—H1···S2^ii^	0.86	2.46	3.2528 (16)	154
N4—H4A···S1^ii^	0.86	2.63	3.4856 (16)	172
C2—H2···S1^iii^	0.93	2.83	3.7492 (19)	168

Symmetry codes: (ii) −*x*+2, −*y*, −*z*+1; (iii) −*x*+2, −*y*, −*z*.
